# Viral Cross-Class Serpin Inhibits Vascular Inflammation and T Lymphocyte Fratricide; A Study in Rodent Models In Vivo and Human Cell Lines In Vitro

**DOI:** 10.1371/journal.pone.0044694

**Published:** 2012-09-26

**Authors:** Kasinath Viswanathan, Ilze Bot, Liying Liu, Erbin Dai, Peter C. Turner, Babajide Togonu-Bickersteth, Jakob Richardson, Jennifer A. Davids, Jennifer M. Williams, Mee Y. Bartee, Hao Chen, Theo J. C. van Berkel, Erik A. L. Biessen, Richard W. Moyer, Alexandra R. Lucas

**Affiliations:** 1 Vascular Biology Research Group, Robarts' Research Institute, London, Canada; 2 Division of Biopharmaceutics, Leiden/Amsterdam Center for Drug Research, Leiden, The Netherlands; 3 University of Maastracht, Maastracht, The Netherlands; 4 Department of Medicine, Divisions of Cardiovascular Medicine and Rheumatology, University of Florida, Gainesville, Florida, United States of America; 5 Department of Molecular Genetics and Microbiology, University of Florida, Gainesville, Florida, United States of America; The University of Texas Health Science Center at San Antonio, United States of America

## Abstract

Poxviruses express highly active inhibitors, including *ser*ine *p*roteinase *in*hibitors (*serpins*), designed to target host immune defense pathways. Recent work has demonstrated clinical efficacy for a secreted, myxomaviral serpin, Serp-1, which targets the thrombotic and thrombolytic proteases, suggesting that other viral serpins may have therapeutic application. Serp-2 and CrmA are intracellular cross-class poxviral serpins, with entirely distinct functions from the Serp-1 protein. Serp-2 and CrmA block the serine protease granzyme B (GzmB) and cysteine proteases, caspases 1 and 8, in apoptotic pathways, but have not been examined for extracellular anti-inflammatory activity. We examined the ability of these cross-class serpins to inhibit plaque growth after arterial damage or transplant and to reduce leukocyte apoptosis. We observed that purified Serp-2, but not CrmA, given as a systemic infusion after angioplasty, transplant, or cuff-compression injury markedly reduced plaque growth in mouse and rat models *in vivo*. Plaque growth was inhibited both locally at sites of surgical trauma, angioplasty or transplant, and systemically at non-injured sites in ApoE-deficient hyperlipidemic mice. With analysis *in vitro* of human cells in culture, Serp-2 selectively inhibited T cell caspase activity and blocked cytotoxic T cell (CTL) mediated killing of T lymphocytes (termed fratricide). Conversely, both Serp-2 and CrmA inhibited monocyte apoptosis. Serp-2 inhibitory activity was significantly compromised either *in vitro* with GzmB antibody or *in vivo* in ApoE/GzmB double knockout mice. ***Conclusions*** The viral cross-class serpin, Serp-2, that targets both apoptotic and inflammatory pathways, reduces vascular inflammation in a GzmB-dependent fashion *in vivo*, and inhibits human T cell apoptosis *in vitro*. These findings indicate that therapies targeting Granzyme B and/or T cell apoptosis may be used to inhibit T lymphocyte apoptosis and inflammation in response to arterial injury.

## Introduction

*Ser*ine *p*rotease *in*hibitors or *serpins* have extensive regulatory actions, moderating thrombotic and immune responses [1], [2]. Poxviruses encode highly active serpins, including a secreted serpin Serp-1, which inhibits extracellular thrombolytic and thrombotic proteases and markedly reduces arterial inflammation and plaque growth in animal models [2]–[4]. This serpin, when injected as a purified protein, also significantly reduces markers of myocardial damage after stent implant in patients with unstable coronary syndromes [5]. These studies suggest that other viral serpins may have therapeutic potential. With the studies reported herein we explore a second class of viral cross-class serpins with different protease targets that block apoptosis and inflammation.

Serp-2, encoded by myxoma [6], [7], and CrmA (cytokine response modifier-A) encoded by cowpox [8], [9] are poxviral cross-class serpins that inhibit the serine protease, granzyme B, and cysteine proteases, caspases 1 and 8. These serpins are purportedly intracellular defense proteins; however, both serpins inhibit pathways with potential extracellular activity. CrmA is a more potent inhibitor *in vitro*, binding caspase 1, caspase 8, and granzyme B (GzmB), with greater inhibition in chicken chorioallantoic membranes [8]-[11]. In contrast, Serp-2 binds caspases 1 and GzmB with lower affinity *in vitro* [6], [7], [10], [11], but has greater effects on viral virulence *in vivo* in rabbits infected with Serp-2 deficient myxomavirus [6]–[8], [11].

Key pathways to cellular apoptosis, also termed programmed cell death, are mediated by serine and cysteine proteases [10], [12]–[18]. Caspases are cysteine proteases, some of which drive intracellular apoptotic pathways, whereas the serine protease GzmB is released by activated T cells into the surrounding medium and inserted into target cells. GzmB initiates apoptosis, either via interaction with perforin or through less defined pathways [13], [16]–[22]. Granzyme B thus has both intracellular and extracellular activities, initiating two-tiered caspase activation in which caspases 3, 7, 8, and Bid (BH3-interacting domain death agonist) play central roles [10], [12], [14], [15]. Granzyme B also cleaves proteases and inhibitors that protect against DNA degradation, specifically topoisomerase, poly (ADP ribose) polymerase (PARP), and inhibitor of caspase-activated deoxyribonuclease (iCAD) [15]. Topoisomerase is part of the DNA repair machinery, PARP releases topoisomerase stalled in the repair process, and iCAD blocks caspase activation of deoxyribonuclease (DNAse).

Apoptosis of endothelial cells, monocytes, and T cells leads to release of pro-inflammatory mediators, creating a cycle of inflammation and cell death. Caspase 1 directly activates interleukin-1beta (IL–1β) and the inflammasome, involved in the macrophage cell death pathway called pyroptosis [23], [24]. In atherosclerotic plaques, increased numbers of apoptotic cells, including T cells, are found at sites of plaque rupture. Monocyte and T lymphocyte invasion, together with endothelial cell dysfunction, are closely linked to atherosclerotic plaque growth and vessel occlusion [16], [25]. Apoptosis induces a pro-thrombotic and pro-inflammatory state in endothelium [13], [16]–[18], [25], while in macrophages and smooth muscle cells [13], [16]–[18] apoptosis is implicated in plaque rupture, the underlying cause for sudden arterial thrombotic occlusion in heart attacks and strokes [16], [17].

While environmental factors such as smoking, high fat or high cholesterol diets, lack of exercise or diabetes can cause initial injury to the arteries, plaques can be found in otherwise healthy individuals at the branching points of arteries, as they have low shear stress and unpredictable blood flow and thus recruit additional inflammatory cells [5], [16]–[18]. Increased numbers of cytotoxic, perforin-positive T lymphocytes are present in inflammatory vascular disease, unstable coronary syndromes, and accelerated transplant vasculopathy [19]–[21], potentially driving cell death. Additionally, activated T cells express CD154 which binds to CD40L present on macrophages and allows for cross-talk and cross-activation of the innate and humoral immune systems. Monocytes secrete cytokines such as interleukin-2 (IL-2) and interferon γ (IFN γ) to alert other lymphocytes to the injury and stimulate them to mature into macrophages and effector T cells [22]. Interference with T cell apoptosis in rats [13] leads to a transplant tolerant state whereas GzmB deficiency in mice reduces transplant vasculopathy in some models [20]. Fas Ligand (FasL) has been reported to either block [26] or to accelerate [27] atheroma development in ApoE-deficient mice. FasL and GzmB are also associated with T cell death induced by other cytotoxic T cells (CTL) changing the balance of T cell subsets {e.g. CD8 T cells, CD4 T helper cells (TH1, TH2, TH17), and CTL} and altering immune responses [28]–[30]. T cell apoptosis may thus contribute toward plaque progression [12], [13], [16], [17], [19]–[22], [26], [27] but the precise role and effects on the balance of different T cell subsets remains only partially defined.

We present here a series of studies examining potential extracellular effects of intracellular cross-class serpins, Serp-2 and CrmA on inflammatory vascular disease in animal models [2]–[4], [31]–[33], with selective analysis of GzmB mediated cellular apoptosis and T cell fratricide.

## Results

### Serp-2 reduces plaque growth in arterial surgical injury models, in vivo

**Figure 1:**
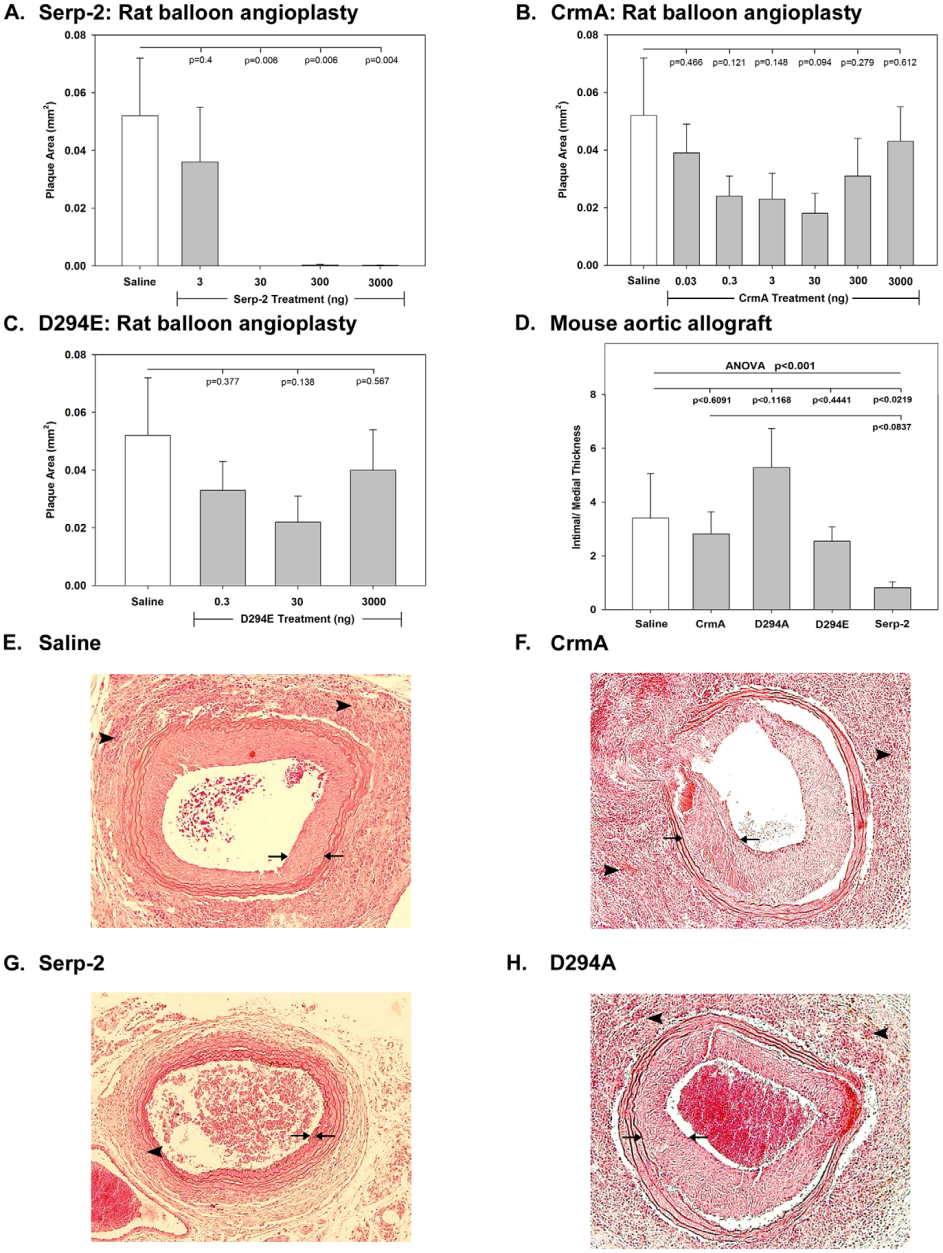
Serp-2 reduces plaque growth in arterial surgery models. Treatment of rat models of arterial injury with viral cross-class serpins demonstrates reduced vasculopathy. Haematoxylin and eosin stained sections of rat iliofemoral arterial sections harvested at 4 weeks and the mean plaque area were measured and presented as mean ± SE. The results demonstrated reduced plaque growth with Serp-2 (6 rats/dose; 3–3000 ng total N = 24) treatment at doses >30 ng (**A**). CrmA (6–12 rats/dose, 0.03–3000 ng; total N = 60) (**B**) and the Serp-2 reactive center loop mutant D294E **(**6 rats/dose, 0.3–3000 ng; total N = 18) treatments (**C)** demonstrated a non-significant trend toward reduced plaque at 30 ng, with no significant inhibition of plaque growth at higher concentrations. (**D**) In the mouse aortic allograft transplant model (PAI-1^−/−^, B6.129S2-*Serpine1^tm1Mlg^* donor to Balb/C recipient) Serp-2 again significantly reduced plaque (p<0.026), whereas CrmA and the D294A and D294E Serp-2 RCL mutants did not reduce plaque (total mice 33). (**E–H**) Five micron thick cross sections of mouse aortic allograft transplants taken from within the transplanted donor aortic section, demonstrate the marked intimal hyperplasia and associated mononuclear cell invasion in the adventitial layers in saline (**E**) or CrmA (**F**) treated mice. Treatment with Serp-2 (**G**) but not Serp-2 D294E mutants (**H**), displayed reduced plaque and inflammation. Arrows bracket intimal plaque limits. Arrowheads point to areas of mononuclear cell invasion. Magnification 100X.

To assess potential extracellular effects of Serp-2 and CrmA on arterial plaque growth, we infused a single dose of individual purified proteins immediately after arterial surgery (**Table** **1**) Two animal models were initially examined; 1) a balloon angioplasty injury model [2], [31], [33] and 2) an aortic transplant model [3], [4]. Saline, Serp-2, CrmA and two Serp-2 mutants were individually assessed after balloon angioplasty (N = 126, **Table 1** **,** **Fig. 1A–C** **)**. Serp-2 treatment (6 rats/dose; total N = 24) significantly reduced plaque growth at doses of 30 ng (0.10 ng/g) or higher (**Fig. 1A**: 3 ng/ p = 0.400; 30 ng/p<0.006; 300 ng/p<0.006; 3000 ng/p<0.004) after mechanical angioplasty injury when compared to saline (N = 6). Treatment with CrmA (12 rats/dose; total N = 60; **Table 1**, **Fig. 1B**: 0.03 ng/p = 0.466; 0.3 ng/p = 0.121; 3 ng/p = 0.148; 30 ng/0.094; 300 ng/p = 0.279 and 3000 ng/p = 0.612) or either of the two active site mutations of Serp-2, D294E (6 rats/dose; N = 18; **Fig. 1C**: 0.3 ng/p = 0.377; 30 ng/p = 0.138 and 3000 ng/p = 0.567) or D294A (6 rats/dose; N = 18; 0.3 ng/p = 0.821; 30 ng/p = 0.076 and 3000 ng/p = 0.623, not shown) showed a trend toward reduced plaque at the 30 ng dose, but failed to inhibit plaque growth at higher concentrations. These results demonstrate that Serp-2 consistently reduced plaque growth in a rat iliofemoral angioplasty model when compared to CrmA, and Serp-2 RCL mutants.

**Table 1:** Animal models.

Strain	Total No.	No. Survival	(%) Survival	Treatment	Strain	Total No.	No. Survival	(%) Survival	Treatment
**STUDY 1A – Rat Iliofemoral Angioplasty: 4wks**					**STUDY 1B – Rat Angioplasty: 0– 72 hrs**				
SD	6	6	100%	Saline	SD	20	20	100%	Saline
SD	6	6	100%	Serp-2 3ng	SD	20	20	100%	Serp-2 300ng
SD	6	6	100%	Serp-2 30ng	SD	20	20	100%	CrmA 300ng
SD	6	6	100%	Serp-2 300ng	SD	20	20	100%	D294A 300ng
SD	6	6	100%	Serp-2 3000ng	SD	20	20	100%	D294E 300ng
SD	6	6	100%	CrmA 0.03ng	**Total**	100	100	100%	
SD	6	6	100%	CrmA 0.03ng					
SD	12	12	100%	CrmA 3ng	**STUDY 2 – Mouse Aortic Allograft**				
SD	12	12	100%	CrmA 30ng	PAI-1 ^−/−^	6	5	83.30%	Saline
SD	12	12	100%	CrmA 300ng	PAI-1 ^−/−^	6	5	83.30%	Serp-2 15µg
SD	12	12	100%	CrmA 3000ng	PAI-1 ^−/−^	9	6	66.70%	CrmA 15µg
SD	6	6	100%	D294A 0.3ng	PAI-1 ^−/−^	5	5	100%	D294A 15µg
SD	6	6	100%	D294A 30ng	PAI-1 ^−/−^	7	5	71.40%	D294E 15µg
SD	6	6	100%	D294A 300ng	ApoE ^−/−^	5	5	100%	Saline
SD	6	6	100%	D294A 3000ng	ApoE ^−/−^	5	5	100%	Serp-2 15µg
SD	6	6	100%	D294E 0.3ng	ApoE ^−/−^	6	5	83.30%	CrmA 15µg
SD	6	6	100%	D294E 30ng	GzmB ^−/−^	6	6	100%	Saline
SD	6	6	100%	D294E 300ng	GzmB ^−/−^	5	5	100%	Serp-2 15µg
SD	6	6	100%	D294E 3000ng	GzmB ^−/−^	6	5	83.30%	CrmA 15µg
**Total**	126	126	100%		GzmB ^−/−^	7	5	71.40%	D294A 15µg
**STUDY 3 – Mouse Carotid Cuff Injury**					GzmB ^−/−^	5	5	100%	D294E 15µg
ApoE ^−/−^	11	11	100%	Saline	ApoE ^−/−^, GzmB ^−/−^	6	5	83.30%	Saline
ApoE ^−/−^	11	11	100%	Serp-2 1.8µg/d	ApoE ^−/−,^ GzmB ^−/−^	6	5	83.30%	Serp-2 15µg
ApoE ^−/−^	11	11	100%	CrmA 240 ng/d	ApoE ^−/−,^ GzmB ^−/−^	6	5	83.30%	CrmA 15µg
**Total**	33	33	100%		**Total**	96	82	85.40%	

SD – Sprague Dawley; ApoE^−/−^ – Apolipoprotein E; GzmB^−/−^ – Granzyme B (B6.129S2–*Gzmbtm1Ley*/J); PAI-1^−/−^ – Plasminogen Activator Inhibitor-1 (B6.129S2-*Serpine1^tm1Mlg^).*

Balloon angioplasty injury predominately induces endothelial denudation with smooth muscle cell proliferation and connective tissue scarring, but with less pronounced inflammation. Therefore, treatment with each serpin was assessed after aortic allograft transplant where greater inflammatory cells responses are detected [2], [3], [4], [31], [33]. Plasminogen activator inhibitor-1 (PAI-1) is a mammalian serpin that regulates thrombolytic proteases and PAI-1^−/−^ aortic allografts have increased inflammatory cell invasion and plaque growth [2], [3], [4], [34]. We therefore examined plaque growth in PAI-1^−/−^ donor to Balb/C recipient mouse aortic allografts (N = 33 total transplants). Serp-2 (N = 6) again reduced plaque growth in transplanted aortic segments [p<0.05 compared to saline (N = 6); p<0.026 compared to CrmA (N = 9)], while CrmA and the two Serp-2 mutants, D294A (N = 5) and D294E (N = 7), did not reduce plaque (**Fig. 1D**). Mutant D294E was predicted to increase inhibitory activity due to preserved negative charge, however, no significant anti-plaque activity was detected with D294E or D294A ; conversely, D294A increased plaque (**Fig. 1D**) when compared to CrmA (p<0.047), D294E (p<0.025), or Serp-2 (P p<0.0005) treatments.

Histological cross sections from aortic allograft transplant (B6.129S2-*Serpine1^tm1Mlg^* PAI-1^−/−^ to Balb/C recipient) with saline treatment (**Fig. 1D, 1E**) or CrmA treatment (**Fig.** **1D, 1F**) displayed rapid plaque growth at 4 weeks with mononuclear cell invasion (**Fig. 1F**, indicated by arrow heads). Serp-2 significantly reduced plaque growth at doses of 1.5 μg (50 ng/g), with reduced inflammatory cell invasion (**Fig. 1D, 1G** **;** plaque thickness demarcated by arrows; p<0.044), whereas Serp-2 mutants D294A and E (**Fig. 1D, 1H**) did not reduce plaque growth.

Serp-2 reduced inflammatory cell invasion in the adventitia (N = 10, 39.53+4.51 cell counts per high power field) when compared to saline (N = 10, 71.43+6.48 cells, p<0.019) and CrmA (N = 10, 77.27+9.59 cells, p<0.007). Conversely, D294A increased inflammatory cell invasion in the intima (N = 6, mean: 25.18 +2.21, p<0.034, saline N = 10, 23.7 +1.90) and decreased it in the adventitia (N = 6, 46.06 +12.99, p<0.0341, saline N = 10, 71.43+6.48), whereas D294E had no significant effects compared to saline.

Serp-2 treatment also reduced plaque in rat aortic transplant models (ACI donor to Lewis recipient, p<0.05, data not shown) and reduced mononuclear cell invasion, when compared to CrmA, in both intimal (p<0.0001, data not shown) and adventitial layers.

These initial studies demonstrate an arterial anti-inflammatory effect for an intracellular viral serpin, Serp-2, in both angioplasty injury as well as aortic allograft transplant models in rodents, when infused into the circulating blood immediately after injury. This effect was specific to Serp-2 and was neither reproduced by another intracellular serpin, CrmA nor by two active site mutants of Serp-2.

### Serp-2 reduces plaque growth in Apolipoprotein E deficient (ApoE^−/−^) mice

**Figure 2:**
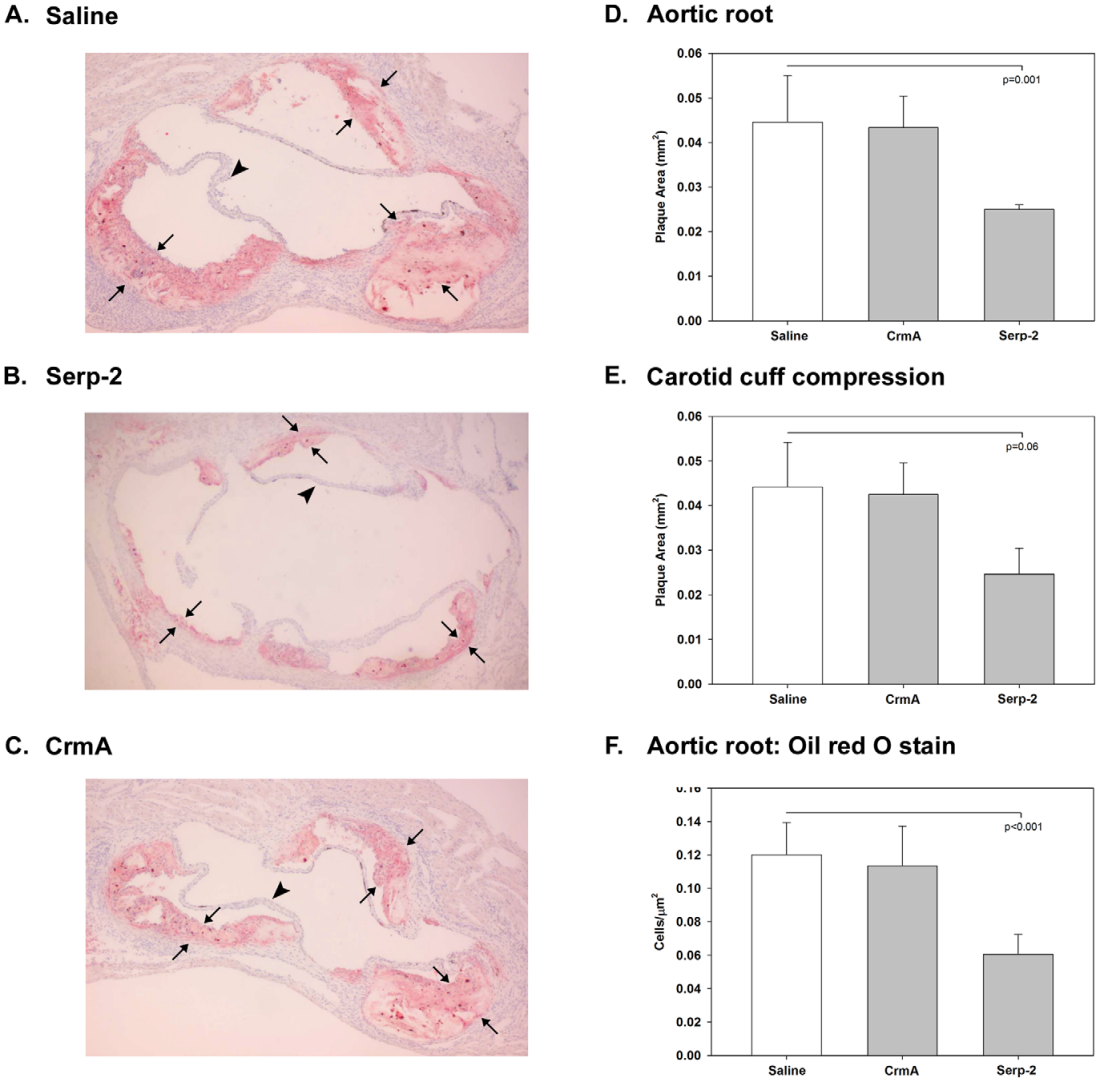
Serp-2 reduces plaque growth in Apolipoprotein E deficient (ApoE^−/−^) mice. Hyperlipidemic ApoE^−/−^ mice were infused with a bolus of Serp-2, CrmA or control saline after carotid cuff compression injury. Histological sections taken at the aortic root, where no surgical injury occurs, and distally at the site of cuff injury were examined. Cross sections taken at the aortic valve level (Oil red O staining) demonstrate plaque growth in saline treated control mice (A) (N = 11). A significant reduction in plaque area is detectable with Serp-2 (**B,** N = 11), but not with CrmA (**C,** N = 11) treatment compared to saline treated controls. Morphometric analysis of plaque area at the aortic root in ApoE^−/−^ mice, where no surgery has been performed, demonstrated that Serp-2 inhibited aortic plaque and macrophage/foam cell invasion (**D**, p<0.001) to a greater extent than at sites of vascular carotid compression surgery in the same model (**E**, P = 0.06). Oil red O staining confirmed a reduction in fatty plaque in the ApoE^−/−^ aortic root (**F**), indicating decreased foam cell/macrophage invasion (p<0.001). Thin arrows bracket intimal plaque limits, larger arrows identify an aortic leaflets, large arrow with open base points to area of fatty, foam cell (macrophage) invasion; Magnification – 100X.

Serpin treatment in hyperlipidemic ApoE^−/−^ mice after carotid cuff compression injury was examined, both at the site of cuff injury and at the aortic root where no surgical injury occurs (**Fig. 2**) [32]. This model provides a means to assess both effects of serpin treatment after arterial surgical injury and also at a site of de novo growth of plaque induced by genetic hyperlipidemia with no arterial surgical injury (e.g. apolipoprotein E deficiency). Serp-2 significantly reduced plaque area in the aortic root (N = 11, p<0.001, **Fig. 2B, 2D**) where there was no surgical injury, but with borderline significance at sites of carotid cuff compression injury (N = 11, p = 0.06, **Fig. 2E**), when compared to saline (**Fig. 2A, 2D**). Plaque reductions with Serp-2 treatment were comparable at both sites, 42% for aortic root versus 44% for the carotid (**Fig. 2D, 2E**), while CrmA treatment (N = 11) had no effect (**Fig. 2C, 2D, 2E**). Compared to saline, plaque lipid content was also significantly reduced on Oil-red O stained sections with Serp-2 treatment, but not with CrmA (**Fig. 2F**). This reduction in lipid-laden cells is similar to the reduction in inflammatory cell invasion seen with Serp-2 treatment in the aortic angioplasty and allograft models.

### Cross-class serpin treatments modify apoptotic responses in vitro

Effects of serpin treatments on cellular apoptotic responses were assessed both at early times after angioplasty injury *in vivo* and *in vitro* in human cell lines. Changes in individual cells in the arterial wall may be masked by analysis of arterial extracts, therefore effects of serpins on apoptosis were examined in individual cell lines in addition to assessing changes in the arterial wall.

At early times post aortic angioplasty injury (12h) in rat arteries, increased fragmented nucleosomes and higher levels of caspase 3, 7, 8, and granzyme B were detected when compared to saline control treatment (p<0.0001). Treatment with individual serpins demonstrated a trend towards reduced caspase and GzmB activity, but no significant reduction was detectable. For instance when DEVDase (caspase 3 and 7) activity was tested, Serp-2 (1.52±0.18; p<0.017), CrmA (1.56±0.18; p<0.0001), D294A (1.8±0.31; p<0.037), or D294E (1.67±0.25; p<0.008) treatment produced significant differences when compared to saline (2.3±0.4), but did not show any difference in activity between Serp-2, Serp-2 mutants, and CrmA treatments.

To test for potential effects on individual cell types associated with arterial inflammation and plaque growth, inhibition of apoptotic responses were measured in HUVEC (human umbilical vein endothelial cells), THP-1 monocytes, and Jurkat T lymphocytes *in vitro*, with and without serpin treatment. Apoptotic responses were induced through three pathways using staurosporine (STS; intrinsic pathway), and camptothecin (CPT; double strand break initiation).

**Figure 3:**
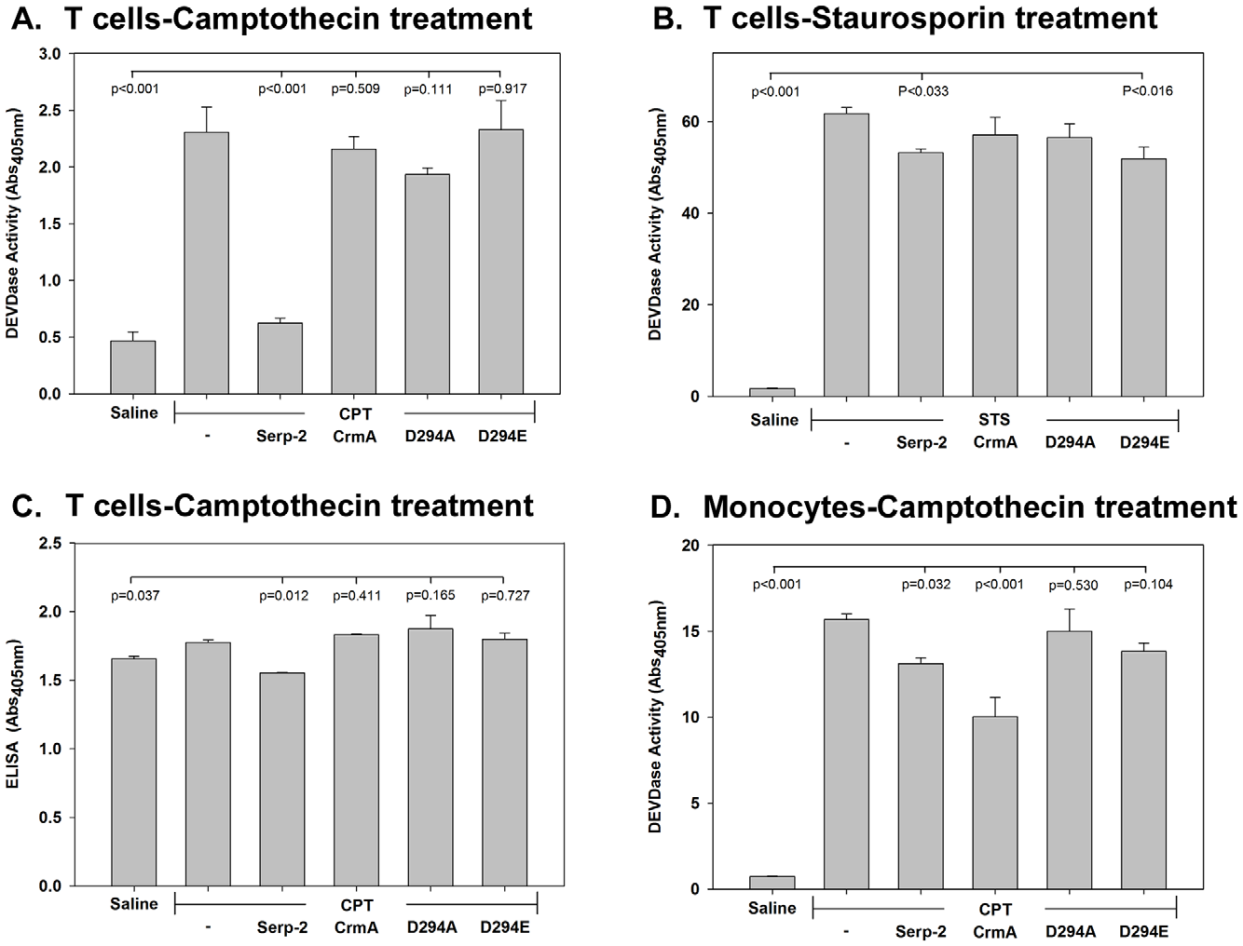
Viral cross-class serpins alter apoptotic responses in T cells and monocytes, *in vitro*. Apoptotic responses were induced in T cells and monocytes using camptothecin or staurosporine. Inhibition of caspase 3 and 7 activity was measured after treatment with Serp-2, CrmA, or the two Serp-2 mutants by analysis of changes in DEVDase activity, with comparison to untreated controls. Serp-2, but not CrmA nor D294A and D294E treatment of Jurkat T cells reduced caspase 3 activity (DEVDase) after camptothecin (CPT) (**A**, p≤0.001) or staurosporine (STS) (**B**, p≤0.033) apoptosis actuator treatment. Cell death in T cells measured as fragmented DNA by ELISA was also blocked in CPT treated T cells (**C**, p≤0.012). Serp-2 (p≤0.032) and CrmA (p≤0.001) both significantly reduced CPT-induced elevations in caspase 3 and 7 activity in monocytes (**D**). The results shown here represent mean ± SE from 3 to 5 replicates for each experiment. Significance was assessed by analysis of variance (ANOVA) with secondary Fishers least significant difference and Mann Whitney analysis.

In T cells, caspase 3 and 7 activity (as measured by DEVDase assay) were significantly increased after camptothecin (**Fig. 3A**, P<0.001) and staurosporine (**Fig. 3B**, P<0.0001) treatment [48]. Granzyme B and caspase 8 (as measured by IEPDase assay) [35] were significantly increased by STS (P<0.0001, **Fig. S1**). Serp-2 reduced caspase 3 and 7 in T cells after camptothecin (**Fig. 3A**, P<0.001) and after staurosporine (**Fig. 3B**, P<0.033) treatment, while CrmA did not alter T cell responses after CPT or STS (**Fig. 3A**). Serp-2 inhibition was more pronounced with CPT treatment (**Fig. 3A**) than after STS (**Fig. 3B**) in T cells. Serp-2 also significantly reduced apoptosis measured by cell death ELISA in T cells after CPT treatment (**Fig. 3C**, p<0.012), while CrmA did not.

In THP-1 monocytes, camptothecin (**Fig. 3D**, p<0.001) and staurosporine (p<0.0009, not shown), both significantly increased caspase 3 and 7 activity, but had little effect on caspase 8 and GzmB (**Fig. S1B**). In monocytes, caspase 3 and 7 were significantly reduced by both Serp-2 (p<0.032) and CrmA (p<0.001) after CPT treatment (**Fig. 3D**), with CrmA producing greater inhibition. Conversely, neither Serp-2 nor CrmA significantly altered GzmB and caspase 8 in THP-1 cells treated with STS (**Fig. S1**).

The Serp-2 mutants, D294A and D294E, did not alter caspase activity in T cells after camptothecin treatment (**Fig. 3A**, p = 0.11), but D294E (although not D294A) did reduce caspase 3, 7, 8 and GzmB with staurosporine (**Fig. 3B**, p<0.016), indicating that the D294E RCL mutant retains some anti-apoptotic activity that differs from endogenous Serp-2. D294A and D294E had no inhibitory activity when tested in THP-1 monocytes after camptothecin or staurosporine treatment (**Fig. 3D**, p = 0.104).

In HUVEC cultures treated with serum deprivation, staurosporine, or camptothecin caspase 3, 7, 8 and GzmB were increased (p<0.001, not shown). Serp-2 and CrmA both significantly reduced caspase 8 after CPT treatment (p<0.0001) in HUVEC, but had no effect on caspase 3 and 7 (not shown). STS-induced apoptosis was not altered by Serp-2, CrmA, D294A, or D294E in HUVEC (not shown). In all cell lines tested after FasL treatment Serp-2, CrmA, and D294A had no inhibitory activity (not shown). The Serp-2 RCL mutant, D294E did, however, reduce caspase 3, 7, 8 and GzmB activity after FasL treatment of T cells (p<0.0005, not shown). Cathepsin K, S, L, V activity in T cells was not affected by serpin treatment (p = 0.386, not shown).

In summary, Serp-2, but not CrmA, inhibited camptothecin- and staurosporine-induced caspase activity in T cells *in vitro*. Both Serp-2 and CrmA inhibited camptothecin-induced caspase activity in monocytes with CrmA displaying greater inhibitory activity in monocytes, suggesting T lymphocytes as a primary target for Serp-2. No differential effects were produced by Serp-2 and CrmA *in vivo* in arterial sections isolated early after angioplasty injury, which may be due to the fact that multiple cell types are assessed in arterial sections.

### Serp-2 reduces T cell apoptotic responses to cytotoxic T lymphocyte (CTL) granzyme B

**Figure 4:**
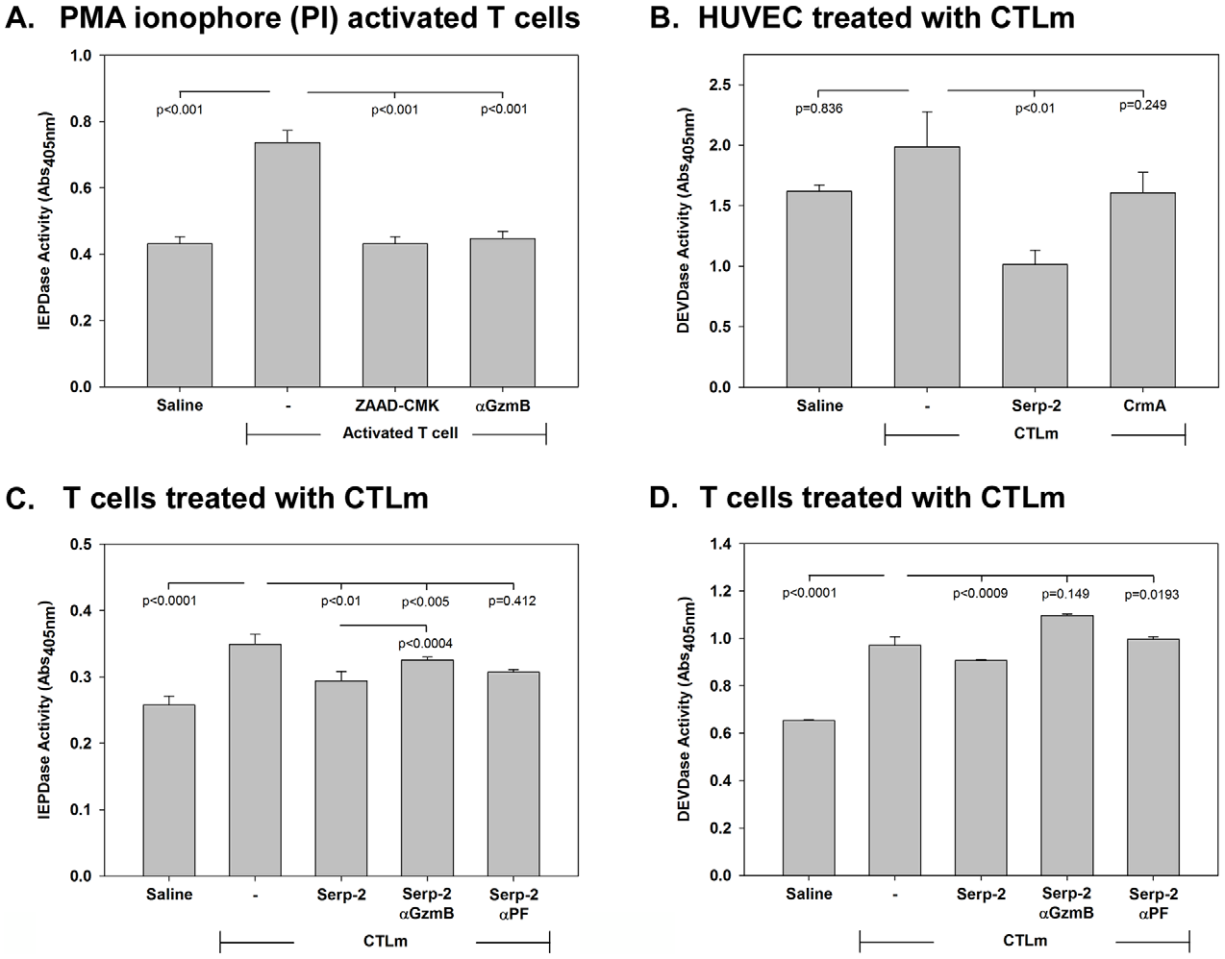
Blockade of granzyme B reduces viral cross-class serpin inhibition of T cell induced T cell apoptosis. Jurkat T cells were treated with PMA and ionophore (PI) and the level of granzyme B expressed was measured by IEPDase assay and caspase 3 and 7 activity by DEVDase assay (**A**). Increased granzyme B (GzmB, p<0.001) secreted by these cells and was inhibited by treating the cells with an intracellular inhibitor of granzyme B, ZAAD-CMK (p<0.001) or anti-granzyme B antibody (p<0.001) (**A**). The medium containing granzyme B from PI treated T cells (CTLm) was applied to naive HUVECs to induce apoptosis (**B**). Treatment with Serp-2, but not CrmA, reduced caspase activity in CTLm treated HUVECs (**B**). The CTLm was also applied to naive T cells in culture to induce apoptosis and increased levels of caspase 3 and granzyme B were observed as IEPDase activity (**C**, p<0.0001) and DEVDase activity (**D**, p<0.0001) respectively. Treatment with Serp-2 reduced both granzyme B (**C**, p<0.01) and caspase 3 (**D**, p<0.0009) activities significantly. Antibody to granzyme B (GzmB) blocked Serp-2 mediated reductions in CTLm induced granzyme B (**C)** when compared to Serp-2 treatment alone (p<0.0004), but with a still significant decrease (p<0.005) when compared to CTLm treatment alone. Antibody to granzyme B also blocked the Serp-2 mediated decrease in caspase 3 (**D**, p<0.149) when compared to CTLm activation. This Serp-2 mediated inhibition of CTLm induced granzyme B activity was also blocked by incubation of cells with antibody to perforin (**C**, p = 0.412) but not the caspase 3 activity (**D**, p<0.0193). The results shown here represent mean ± SE from 3 to 5 replicates for each experiment. Significance was assessed by analysis of variance (ANOVA) with secondary Fishers least significant difference and Mann Whitney analysis.

Activated T cells (TH1) and cytotoxic T lymphocytes/ natural killer (CTL/NK) cells release GzmB into the surrounding medium, initiating death responses in other cells, and also in T lymphocytes [12], [14], [15]. The role of GzmB in Serp-2 mediated anti-apoptotic activity was assessed using medium from T cells activated to a CTL-like state with phorbol myristic acid (PMA) and ionophore [12], [14]. These activated CTL-like cells express and secrete increased GzmB. Naive HUVEC, THP-1, and Jurkat cells were then treated with medium from the CTL-like T cells (CTLm) with and without serpin treatments (**Fig. 4** **, Fig. S1**).

Increased secreted extra-cellular GzmB was detected in PMA and ionophore treated Jurkat cultures (**Fig. 4A**). Both anti-GzmB antibodies and the tetrapeptide ZAAD – Chloromethylketone (ZAAD-CMK), a chemical intracellular inhibitor of GzmB, reduced granzyme B and caspase 8 activity (IEPDase) in these CTL cultures (**Fig. 4A**, P<0.001).

IEPDase (GzmB and caspase 8 p<0.0001) activity and DEVDase (caspase 3 and 7 p<0.0001) activity were increased significantly in T cells treated with CTL medium (**Fig. 4C, 4D**), but not significantly in CTLm treated HUVECs (**Fig. 4B**, p = 0.836) nor monocytes (not shown) [35], [36]. Serp-2, reduced caspase 3 and 7 (**Fig. 4B**, p<0.01) and caspase 8 (not shown, p<0.01) in HUVEC cultures, but not in THP-1 (not shown). CrmA did not decrease caspase activity in HUVEC cultures treated with CTLm (p = 0.249, **Fig. 4B**).

In T cells treated with CTLm, Serp-2 produced a significant reduction in CTLm-mediated increases in caspase 8/GzmB (p<0.01, **Fig. 4C**) and in caspase 3/7 (p<0.0009, **Fig. 4D**). Concomitant treatment with antibody to GzmB or perforin blocked Serp-2 inhibition of caspase 8/GzmB (**Fig. 4D**, p<0.0004 compared to Serp-2 and CTLm treatment for GzmB antibody, p = 0.412 for perforin antibody compared to CTLm alone) and caspase 3/7 activity (**Fig. 4D**, p = 0.149 compared to CTLm treatment alone, p<0.0193 for perforin antibody compared to CTLm treatment alone).

### Serp-2 binding to T cells is reduced with granzyme B inhibition

**Figure 5:**
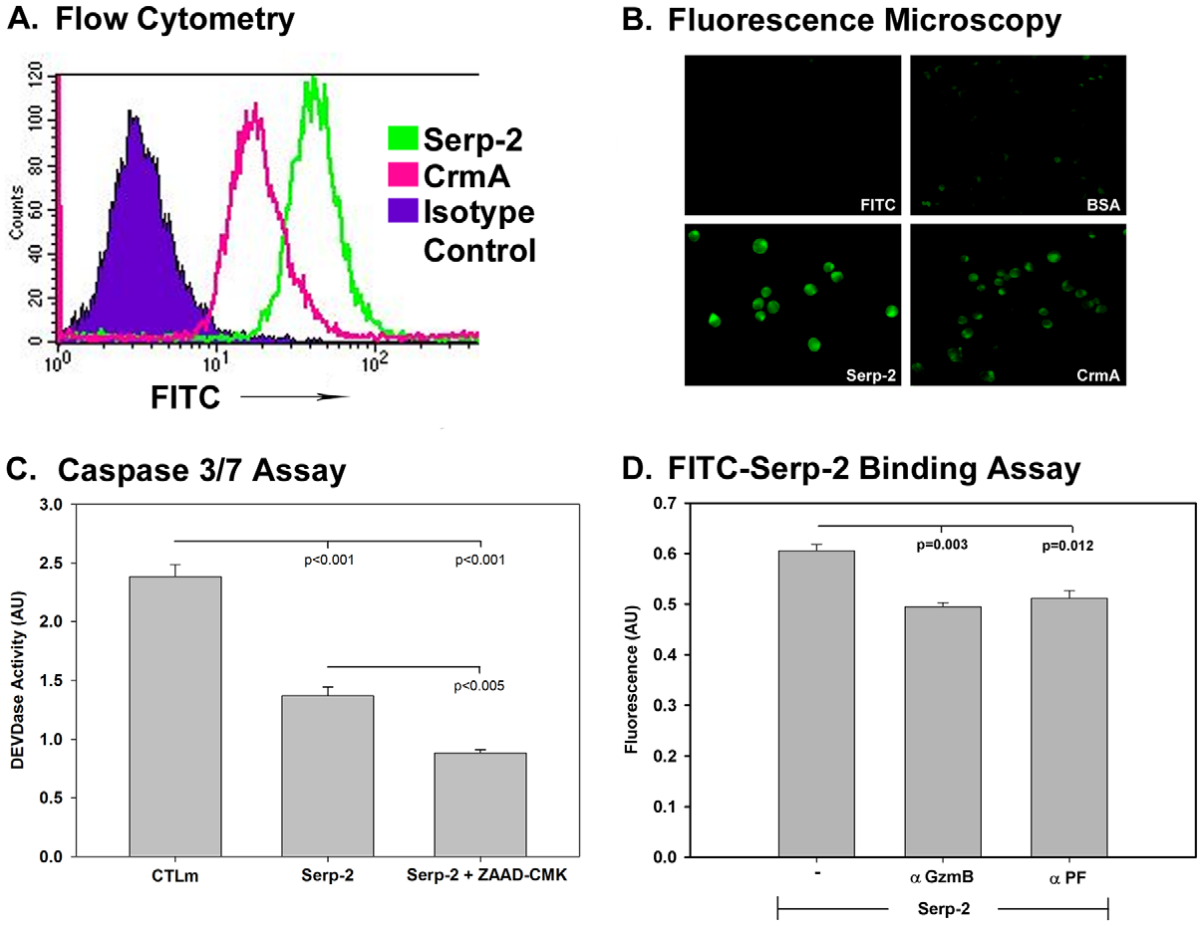
Serp-2 binds T cells *in vitro* with greater affinity than CrmA. Jurkat T cells were treated with FITC labeled Serp-2 or CrmA and binding/ association of these viral proteins with T cells was analyzed using Flow cytometry (FACS) analysis (**A**) and fluorescence microscopy (**B**, Magnification 10X). Extracellular, surface FACS analysis shows both Serp-2 and CrmA bind to the T cell surface (**A**). This observation was also supported by the fluorescent microscopic analysis (**B**). Intracellular ZAAD-CMK granzyme B inhibitor decreased Serp-2 mediated inhibition of caspase 3 activity in response to treatment with CTLm (cytotoxic-like T cell medium) (**C**, p<0.005). Treatment with antibody to granzyme B (α GzmB) or perforin (α PF) partially blocked Serp-2 binding (**D**, p<0.003 and p<0.012, respectively). The results represent mean ± SE from 3 to 5 replicates for each experiment. Significance was assessed by analysis of variance (ANOVA) with secondary Fishers least significant difference and Mann Whitney analysis.

To determine whether Serp-2 or CrmA binds to T cells, FITC-labeled Serp-2 and CrmA binding was measured by flow cytometry (**Fig. 5A**) and fluorescence microscopy (**Fig. 5B**). In these studies Serp-2 displayed a greater binding affinity than CrmA (**Fig. 5A, 5B**) for T cells *in vitro* and mouse peripheral circulating lymphocytes *in vivo* (not shown). Serp-2 significantly reduced caspases 3/7 activity in CTLm treated T cells (**Fig. 5C**, p<0.001) and treatment with the GzmB inhibitor ZAAD-CMK, which predominately inhibits intracellular granzyme B, further increased Serp-2-mediated inhibition of caspases, indicating that Serp-2 actions may be predominately extra-cellular (**Fig. 5C**, p<0.005). The uptake of FITC-labeled Serp-2 into Jurkat T cells, as measured by soluble lysate content, was also reduced after treatment with antibody to GzmB (**Fig. 5D**, p<0.003) or perforin (**Fig. 5D**, p<0.012), further supporting a role for Serp-2 binding to GzmB and inhibiting of T cell responses.

### Serp-2 inhibition of aortic plaque is reduced in granzyme B knockout aortic allografts

**Figure 6:**
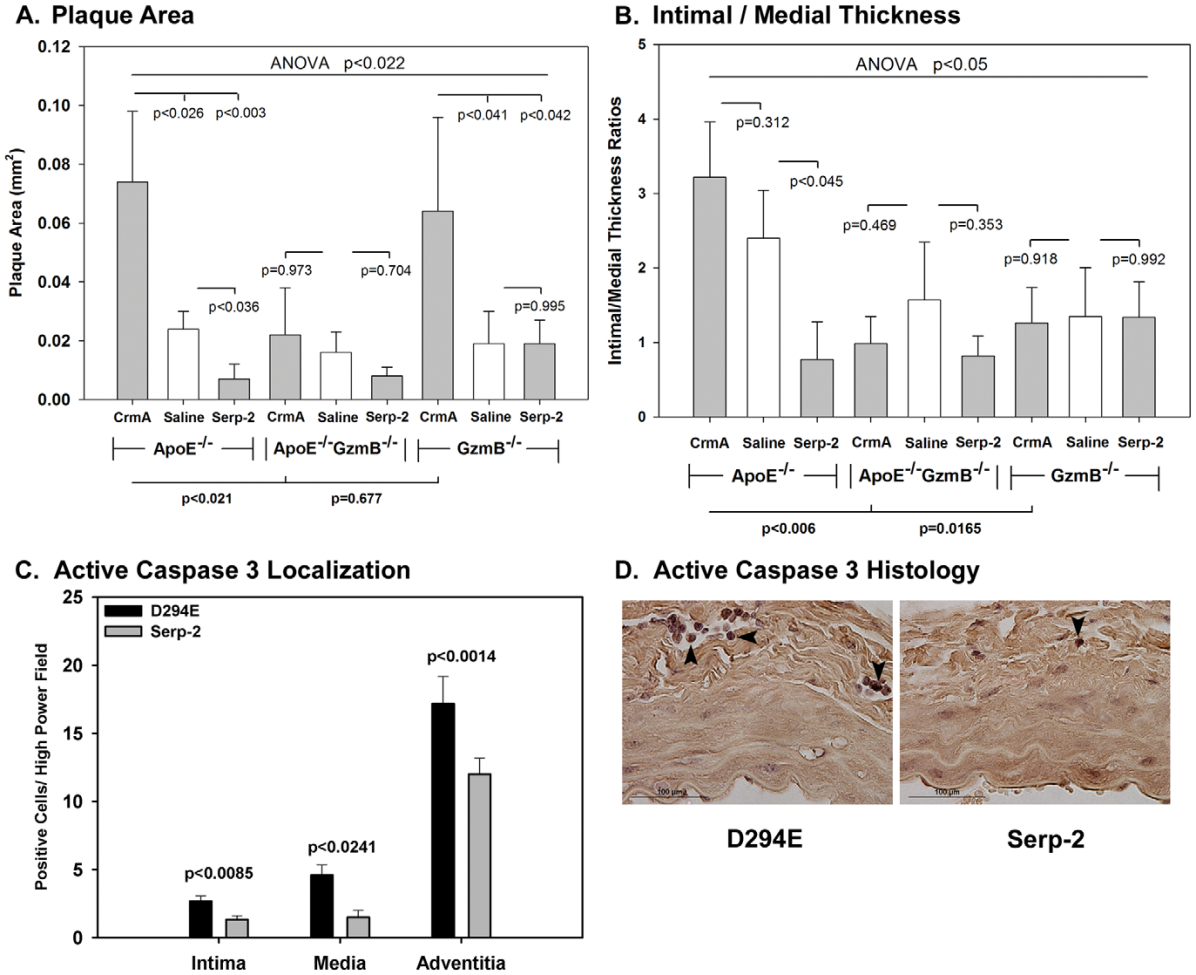
Granzyme B deficiency (GzmB^−/−^) in donor aorta interferes with Serp-2 inhibition of plaque growth after aortic allograft transplant. ApoE^−/−^ (C57Bl/6) donor aortic allograft transplant into Balb/C recipient mice (N = 16) induced plaque growth at 4****weeks follow up as measured by plaque area (**A**) and intimal to medial thickness (IMT) ratios (**B**). Serp-2 treatment significantly reduced plaque area (**A**, p<0.036) and IMT ratios (**B**, p<0.045) when compared to saline treatment. CrmA treatment markedly increased plaque area (**A**, p<0.026) and non-significantly increased IMT ratios (**B**, p = 0.312). Saline treated ApoE^−/−^ GzmB^−/−^ DKO allografts (N = 18) had non-significant reductions in plaque area and IMT when compared to ApoE^−/−^ donor allografts (**A, B**). CrmA treated ApoE^−/−^ GzmB^−/−^ DKO allografts had significantly reduced plaque area (A, p<0.021) and IMT (**B**, p<0.006) when compared to CrmA treated ApoE^−/−^ donor allografts. Serp-2 no longer reduced plaque area (**A**) or IMT (**B**) in either GzmB^−/−^ allografts (p = 0.995 for plaque area and p = 0.992 for IMT) or ApoE^−/−^ GzmB^−/−^ DKO (p = 0.704 for plaque area and p = 0.353 for IMT). Serp-2 but not Serp-2 mutant D294E significantly reduced caspase 3 staining in all three arterial layers (Fig****6C, p<0.024) in PAI-1^−/−^ mice 4****weeks post-aortic transplant. Immunostained sections for caspase 3 illustrate reduced staining in mononuclear cells in Serp-2 treated PAI-1^−/−^ aortic transplants when compared toD294E treatment (Fig****6D, Mag 400X).

We postulated that Serp-2 mediates extracellular anti-inflammatory and anti-atherogenic activity via selective targeting of granzyme B (GzmB). Plaque growth in GzmB^−/−^ single and ApoE^−/−^, GzmB^−/−^ double knockout (ApoE^−/−^, GzmB^−/−^ DKO) aortic allografts was assessed with and without Serp-2 or CrmA treatment. Donor ApoE^−/−^GzmB^−/−^ DKO aortic allografts (N = 18) were compared with ApoE^−/−^ (N = 16) and GzmB^−/−^ (N = 29) allografts (B6 into C57Bl/6 background). ApoE^−/−^ hyperlipidemic mice are expected to have greater *de novo* plaque buildup and thus are predicted to enhance the capacity to detect changes / reductions in plaque growth in GzmB deficient mice. GzmB deficiency has the potential to reduce baseline plaque and thus prevent detection of a further reduction in plaque size after serpin treatment. ApoE^−/−^GzmB^−/−^ DKO and single GzmB^−/−^ or single ApoE^−/−^ knockout allografts were therefore assessed for differences in plaque production. In our model, 4 weeks post-transplantation, saline treated ApoE^−/−^GzmB^−/−^ DKO allografts had a significant trend toward reduced plaque area (**Fig. 6A**) and IMT (**Fig. 6B**) when compared to saline treated ApoE^−/−^ allografts (**Fig. 6A**), suggesting that GzmB deficiency in donors has variable effects on plaque growth. At 4 weeks follow up Serp-2 significantly reduced plaque area (**Fig. 6A**, p<0.036) and intimal to medial thickness (IMT) ratios (**Fig. 6B**, p<0.045) in the ApoE^−/−^ allografts. Serp-2 no longer significantly reduced plaque area **(** **Fig. 6A** **)** or IMT ratios (**Fig. 6B**) in GzmB^−/−^ (p = 0.995 for plaque area and p = 0.992 for IMT) or in ApoE^−/−^GzmB^−/−^ DKO allografts (p = 0.704 for plaque area, p = 0.353 for IMT). Conversely, CrmA significantly increased plaque area in ApoE^−/−^ donor allografts (**Fig. 6A**, P<0.026). CrmA increased plaque area in GzmB^−/−^ (p*<*0.041), but not in ApoE^−/−^GzmB^−/−^ DKO (p = 0.973) allografts (**Fig. 6A**) and had no significant effect on IMT (**Fig. 6B**). The increase in plaque area detected with CrmA treatment in ApoE^−/−^ donor allografts (**Fig. 6A**, p<0.026) was significantly reduced in CrmA treated ApoE^−/−^GzmB^−/−^ DKO allografts (**Fig. 6A, 6B**, p<0.021 for plaque area and p<0.006 for IMT). Serp-2, but not the mutant D294E protein, was able to reduce the amount of active Caspase 3 staining in cross sections of mouse aorta 4****weeks after transplant injury **(** **Fig. 6C** **).** Reduced staining was visible in mononuclear cells in the adventitia (p<0.024) for Serp-2 treatment when compared to the less active D294E mutant (**Fig. 6D** **)**.

These studies support GzmB as one of the central targets for Serp-2 mediated anti-inflammatory and anti-atherogenic activity.

### Serp-2 reduces early apoptosis in invading inflammatory cells after aortic allograft transplant

**Figure 7:**
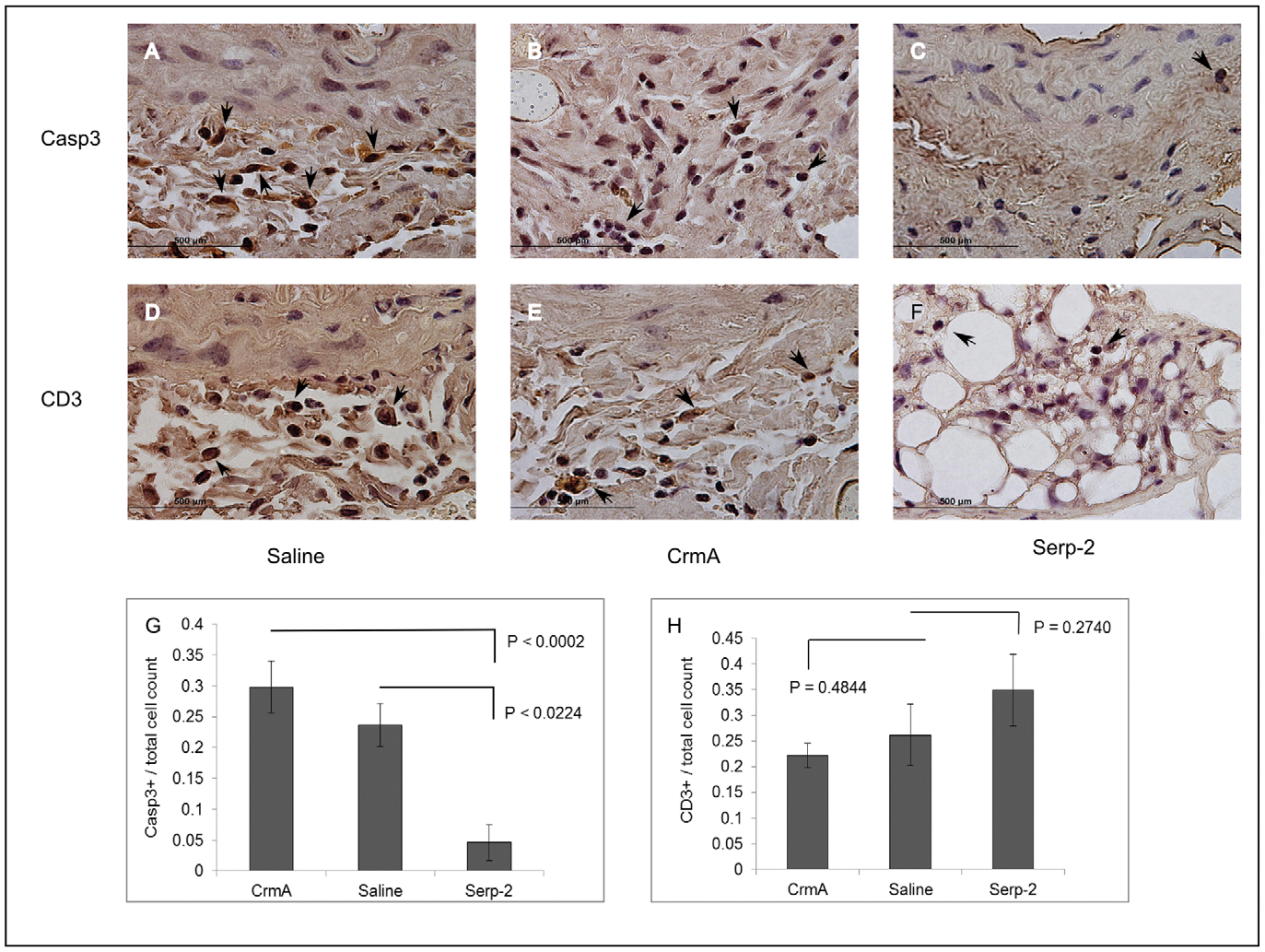
CD3 and active Caspase 3 populations 72 hrs after mouse aortic allograft. C57Bl/6 donor aortic allografts were transplanted into Balb/C recipient mice (N = 3 per treatment) and followed up at 72****hrs. Compared to saline, Serp-2 but not CrmA treatment reduced caspase 3 activity (panels A-C,G; p<0.0224). Neither protein treatment significantly reduced CD3+ T cells (panels D-F,H).

Early changes in T cell and macrophage responses can initiate inflammatory responses that drive plaque development and plaque growth at later times in arterial plaque growth and disease. To assess effects of Serp-2 and CrmA on inflammatory T cell responses *in vivo* in a mouse model, we examined markers for T cell invasion and apoptosis in aortic allograft transplants in mice at early 72 hour follow up (**Fig. 7**). Serp-2 treatment (**Fig. 7**) induced no significant T lymphocyte responses at 72 hrs follow up, although there is a minor trend toward an increase in CD3 positive T cells. On immunohistochemical staining, however, Serp-2 but not CrmA, markedly reduced inflammatory cell apoptosis (**Fig. 7G** **,** p<0.0002).

## Discussion

Many cell types are associated with atherosclerotic plaque growth. Injury to the arterial wall is believed to cause endothelial cell dysfunction and activation of inflammatory cells, specifically monocytes that transform into macrophages, T lymphocytes as well as smooth muscle cells, and other cells types such as mast cells, neutrophils and even B lymphocytes. Damage to the arterial wall and loss of supporting connective tissue can additionally cause programmed cell death or apoptosis, which can lead to release of increased levels of inflammatory cytokines. Activated or dysfunctional T cells can also induce transformation of other cells to a suicidal or apoptotic state. These initial changes in inflammatory cell responses are believed to then drive further damage to the arterial wall and cause intimal plaque growth and arterial narrowing.

It is evident that there are multiple factors that drive plaque growth with some known shared or common pathways. We elected to assess the effect of an anti-apoptotic serpin, as the apoptotic pathways are becoming recognized as a driving force in arterial injury responses and inflammation. Apoptosis in endothelial and in macrophage cells has been reported, as has apoptosis in SMC and T cells in plaque development. However, apoptosis altering the many T cell sub-populations remains poorly defined. It is not known whether interruption of apoptotic responses will alter plaque development and whether this applies to the wide range of arterial injury states that can cause plaque formation.

It is for this reason we have elected to assess plaque growth and responses to the viral anti-inflammatory serpin, Serp-2, in a range of models to determine whether the effects of this protein will be evident in different animal models of arterial disease in order to assess whether this will be of more widespread potential interest. Furthermore, in order to better isolate the contributions of individual cellular subpopulations, individual serpins were tested on human cell lines. Activated or dysfunctional T cells can also induce apoptosis of endothelial cells and monocytes/ macrophages, among other cell types; in the plaque, this leads to an increased release of cytokines and activate thrombolytic serine proteases tissue- and urokinase-type plasminogen activators (tPA and uPA, respectively) and the matrix metalloproteases (MMPs), which breakdown collagen and elastin, weakening the plaque's fibrotic cap [45]–[47]. In addition to these activated and apoptotic cells, the newly exposed necrotic core and eroded cap structure also activate leukocytes and may initiate plaque rupture, subsequent thrombus formation, leading to heart attacks and strokes. Through a series of complex cross-talk and feedback mechanisms, the serine proteases in the coagulation and fibrinolytic pathways interact on many levels with the inflammatory and apoptotic responses and vice versa.

It is evident that there are multiple factors that drive plaque growth with some known shared or common pathways. We elected to assess the effect of an anti-apoptotic serpin, as the apoptotic pathways are now becoming recognized as a driving force in arterial injury responses and inflammation [48]. Apoptosis in endothelial cells and in macrophage has been reported as has apoptosis in SMC and T cells in plaque development. However, apoptosis altering the many T cell sub-populations remains poorly defined. It is not known whether interruption of apoptotic responses will alter plaque development and whether this applies to the wide range of arterial injury states that can cause plaque formation. For this reason, we have elected to assess plaque growth and responses to the viral anti-inflammatory serpin, Serp-2, in a range of models to determine whether the effects of this protein will be evident in different animal models of arterial disease. Furthermore, in order to better tease apart the contributions of individual cellular subpopulations, individual serpins were tested on human cell lines.

Intravenous infusion of Serp-2, a reputed intracellular myxomaviral cross-class serpin, effectively inhibited plaque growth in a series of animal models of vascular disease (**Figs. 1** **,** **2**) irrespective of the model being vascular surgery based or hyperlipidemic mice. These studies demonstrate marked extracellular, GzmB-dependent inhibitory actions for Serp-2, previously thought to function in a predominantly intracellular capacity. This inhibitory activity was unique to Serp-2; the cowpox viral serpin, CrmA and two Serp-2 active site mutants were inactive in these models.

Serp-2 blockade of plaque growth in donor aortic allografts was absent when the transplanted tissue was from GzmB^−/−^ donors. Local deficiency of GzmB was not sufficient to reduce plaque growth in donor allografts treated with saline, but did block increased plaque in ApoE^−/−^ mice treated with CrmA. Thus, granzyme B may have greater effects on vascular disease when active locally rather than systemically during inflammatory cell responses. Although Serp-2 was infused systemically, the loss of activity in donor allografts from knockout mice suggests that Serp-2 acts locally on donor aorta after infusion (**Fig. 6**). *In vitro* studies suggest that Serp-2 specifically inhibits T cell apoptosis. Further work using isografts, GzmB^−/−^ transplant recipients, and caspase 1 deficient transplants is needed to assess and contrast the roles of systemic GzmB and caspase 1 in allograft vasculopathy and as a target for Serp-2.

To try to separate out the effects of different cell lineages found in plaques, the effects of the serpins on apoptosis-induced cell lines were examined. Serp-2 bound to T cells *in vitro* in culture and selectively inhibited caspase 3/7 in camptothecin (CPT)-treated Jurkat T cells (**Figs. 3** **and** **4**) and was dependent upon GzmB and perforin (**Figs. 5** **and** **6**). Additionally, Serp-2 but not Serp-2 D294E was able to reduce levels of active caspase 3 in ApoE and ApoE/GzmB knockout mouse aortic cross-sections after transplant (**Fig. 6**). This Serp-2 mediated reduction for apoptosis was substantiated when comparing Serp-2 to CrmA and saline 72hrs after C57Bl/6 aortic transplant into Balb/C mice (**Fig. 7A–C, 7H**). Based upon these studies we postulate that Serp-2 decreases T cell mediated apoptosis, inducing a generalized reduction in vascular inflammation.

The extracellular activity for this viral serpin is predicted to begin with binding to GzmB. GzmB mediates apoptosis upon release from T cells and can also induce apoptosis in other T cells. Many viral proteins have multiple functions [2], [8], [31] and expanded actions of these cross-class serpins upon release from infected cells is predicted [2]–[9], [31]–[33]. This inhibitory activity is present either with camptothecin treatment of T cells or after treatment with CTL medium from PMA and ionophore treated T cells, containing granzyme B. Serp-2 may thus either bind GzmB outside the cell or may be internalized via perforin pathways. The GzmB inhibitor ZAAD-CMK, which is cell membrane permeable and inhibits GzmB inside the cell, further increased Serp-2 inhibition of caspase activity indicating that Serp-2 actions are extra-cellular. Serp-2 inhibition of camptothecin-mediated apoptosis is consistent with GzmB inhibition.

Many viral proteins are also reported to derive functions through mimicking mammalian genes, as well as the converse. Two mammalian serpins, murine serine protease inhibitor 6 (SPI-6) [36] and human protease inhibitor 9 (PI-9) [37] target GzmB and protect cells from CTL-induced apoptosis. Serp-2 protein may thus mimic this mammalian serpin pathway, hindering T cell apoptosis and inflammation, e.g. T cell fratricide. We have not yet, however, determined whether selected T cell subsets are targeted by Serp-2 protection. It is undeniable that Serp-2 is protecting both T cells and other lineages from GzmB mediated apoptosis (**Fig. 4**) *in vitro*.

GzmB mediates DNA degradation, interfering with DNA repair responses. Topoisomerase, iCAD, and PARP are involved in DNA damage repair and are GzmB substrates. Camptothecin binds topoisomerase I, an enzyme class that alters DNA topography, interfering with DNA re-ligation [38] and creating persistent DNA breaks. Inhibition of topoisomerase also leads to caspase activation [37]. Poly (ADP ribosylation, PAR) is a post-translational modification driven by the PAR polymerase-1 (PARP-1) that reactivates topoisomerase complexes, preventing further damage [39] and is also a transcription initiation factor for NFκB [40]. Once cleaved, iCAD no longer inhibits caspase activated DNAse permitting DNA degradation [41]. Serp-2 mediated inhibition of GzmB or inhibition of secondary induction of caspase 3 may alter the balance between DNA repair and damage in T cells [42]–[44].

We conclude that Serp-2, a viral anti-apoptotic cross-class serpin, has the potential to inhibit arterial vascular disease progression in animal models through inhibition of GzmB-dependent T cell apoptosis. Granzyme B-inhibition of T cell fratricide may represent a potential new target for intervention in inflammation-based disease. Serp-2 inhibition is generalized, with expanded inhibitory function for plaque growth in hyperlipidemic ApoE^−/−^ mice and after arterial surgery, indicative of blockade of central regulatory pathways.

## Materials and Methods

### Animal Models

All research protocols and general animal care were approved by the Robarts' Research Institute, University of Western Ontario, London, Canada, laboratory animal ethics committee and the University of Florida, Gainesville, USA, Institutional Animal Care Committee, (IACUC, Protocol number – 102004234) and conformed to national guidelines. Effects of each serpin on plaque growth were assessed in three animal models; 1} angioplasty injury in 250–300 g male Sprague Dawley rats (SD, Charles River Laboratories, Wilmington, Mass USA; N = 126) [2], [31], [33], 2} aortic allograft transplant from inbred 250–300 g ACI to Lewis rats (N = 60) [2], [31], as well as C57Bl/6 to Balb/C mice (N = 96) [2], [3], [4], [31] (Charles River Laboratories), and 3} carotid cuff compression injury in 12–14 week old C57Bl/6 ApoE^−/−^ mice (N = 33) [32] (TNPO-PG, Leiden, the Netherlands) with all surgeries performed as previously described (**Table 1**). In a second study, ApoE^−/−^ (N = 15), GzmB^−/−^ (B6.129S2-Gzmbtm1Ley/J; N = 15), PAI-1^−/−^ (B6.129S2-*Serpine1^tm1Mlg^*; N = 14) (The Jackson Laboratory, Bar Harbor, Maine) as well as GzmB^−/−^/ApoE^−/−^ DKO mice (C57Bl/6 background, N = 15) used in aortic allograft transplant studies [2]–[4]. GzmB^−/−^/ApoE^−/−^ DKO mice (C57Bl/6 background) were generated at the Breeding Facility at the University of Florida from ApoE and GzmB single knockout mice purchased from Jackson Laboratories and tested for homozygous double knockout status before being used in experiments. All research protocols and general animal care were approved by University laboratory animal ethics and conformed to national guidelines. All surgeries were performed under general anesthetic, 6.5****mg per 100****g body weight Somnotrol (MTC Pharmaceuticals, Cambridge, Canada) intra-muscular injection for rats [2]–[4], [31], [33] and subcutaneous 60****mg/kg ketamine (Eurovet Animal Health), 1.26****mg/kg Fentanyl, and 2.0****mg/kg fluanisone (Janssen Animal Health) for carotid cuff placement in ApoE^−/−l^ mice [32].

Viral serpins were infused intravenously (i.v.) immediately after surgery in rats at doses of 0.3 ng – 3000****ng/rat (0.001–10****ng/g), with follow up at 4 weeks (Table****1). Daily subcutaneous injections of saline, CrmA (240****ng/mouse/day, 12****ng/g/day) and Serp2 (1800****ng/mouse/day, 90****ng/g/day) were started two weeks after collar placement in ApoE^−/−^ mice and continued for 4****weeks until sacrifice. ^125^I labeled CrmA and Serp-2 were injected on the first day and the last two days in two ApoE^−/−^ mice detecting serum concentrations of 0.16****nM for CrmA and 1.72****nM for Serp-2. For the mouse aortic transplant studies a single i.v. injection (15****µg/ mouse; 500****ng/g) of Serp-2, CrmA, or D294A or D294E mutants was administered immediately after allograft surgery. A separate group of 100 rats had angioplasty injury with 300****ng of each serpin by i.v. injection for early follow up at 0, 12, or 72****hours to assess early apoptotic pathway enzyme activity (6 animals/treatment group; Table****1). There were no deaths after angioplasty or aortic transplant in the rats, 14 mice died after aortic transplant, and one mouse died during placement of the carotid cuff. In the mouse aortic transplant model two mice died after treatment with Serp-2, 5 after CrmA, 2 after D294A, 2 after D294E, and 2 after saline treatment with overall survival of 85.4% (p = NS). Body weight was measured weekly and mice were checked daily for signs of distress and necessity for analgesic.

### Histological, Morphometric, and Fluorescence Analysis

At the designated study end (4****weeks for plaque analysis and 0–72****hours for protease activity or apoptosis) rats and mice were sacrificed with Euthanyl (Bimenda MTC Animal Health Company, Cambridge, Ontario, Canada). For mouse and rat angioplasty and allograft transplant models, arterial sections were fixed, processed, paraffin embedded, and cut into 5 µm sections (2–3 sections per site) for histological analysis, as previously described [2]–[4], [31]–[34]. For the ApoE^−/−^ mice with carotid cuff compression, the aortic valve area (10 µm sections throughout the valve area) and the carotid artery from the bifurcation through the site of cuff compression (5 µm sections at 25 µm intervals) were assessed. Sections were stained with Haematoxylin/ Eosin, Trichrome and Oil Red O for analysis of plaque area, thickness, and invading mononuclear cells, as previously described [2]–[4], [31]–[33]. Plaque area as well as intimal thickness and medial thickness were measured by morphometric analysis via the Empix Northern Eclipse trace application program (Mississauga, ON, Canada) on images captured by a video camera (Olympus, Orangeburg, NY, USA) attached to and calibrated to the Olympus microscope objective. The mean total cross-sectional area of the intima as well as diameter of the intima and media were calculated for each arterial section. For immunohistochemistry, T cells were labeled with rabbit anti-mouse CD3 antibody, both then labeled with secondary goat anti-rabbit antibody (CD3; Cat # AB5690, Secondary anti-rabbit; Cat# AB80437. Abcam, Cambridge, MA, USA). For Caspase 3 staining, sections were incubated with anti-caspase 3 polyclonal antibody (Cat# AB3623 1∶20) as primary antibody with secondary rabbit specific-HRP conjugated antibody (Cat # AB80437). Either numbers of positively stained cells in three high power fields in the intimal, medial, and adventitial layers were counted and the mean calculated for each specimen or, when fewer cells were detected, (displayed in earlier follow up times) cell counts for positive staining in all three layers were averaged.

For spectroscopic analysis of Serp-2 and CrmA binding, 1×10^6^ cells/mL were treated with 1ug/mL of FITC-labeled protein for two hours, lysed with cell lysis buffer, and fluorescence emission at 525 nm quantified during excitation at 485****nm. For fluorescence microscopy, cells treated with Serp-2 FITC for 2 hrs at 4°C, then fixed with 2% formaldehyde, mounted with 10% glycerol mounting solution and viewed with a Zeiss fluorescent microscope as previously described [4]. For FACS analysis, 1×10^6^ cells/mL were treated with 1 µg/mL of FITC labeled Serp-2 or CrmA for two hours and run on FACS (FACS Calibur, BD Falcon) acquiring data for 20,000 events with three replicates (Cell Quest data analysis program). To measure uptake of Serp-2, Jurkat T cells were treated with PMA and ionophore to stimulate granzyme B production, then FITC-labeled Serp-2 was added to the activated cells and incubated. Cells were lysed and the membrane and insoluble fractions were separated by centrifugation. FITC-Serp-2 presence was quantitated by absorbance at 525 nm (Fluorscan) measurements for both fractions.

### Cell culture

Human umbilical vein endothelial cells (HUVEC, CC-2519 Clonetics, Walkersville, MD, passages 2–5), THP-1 cells (American Type Culture Collection, Rockville, MD, USA, ATCC TIB-202), or Jurkat cells (E6.1 clone, ATCC TIB-152) (0.5 – 1.0 ×10^6^ cells/ml) were incubated with saline control, one of the apoptosis inducing agents (3 ng/ml membrane bound FasL, 0.5****µM STS, or 2 –10****µM CPT) together with individual serpins (500 ng/10^6^ cells/ml) [4]. Media was supplemented with 10% Fetal Bovine Serum (Invitrogen Canada Inc., Burlington, ON), Penicillin (100 units/ml), and Streptomycin (100****µg/ml, Gibco BRL).

### Source and Purification of Serp-2, CrmA, D294A, and D294E

All serpins were His-tagged at the amino-terminus, expressed in vaccinia/T7 vector in HeLa cells (Dr Richard Moyer, University of Florida, Gainesville, USA) [6], and purified by immobilized metal affinity using His-Bind resin (Novagen) [2]–[8], [31], [33]. The D294A protein is a site-directed mutant of Serp-2 with P1 Aspartate 294 changed to Alanine to inactivate the serpin, while the D294E protein has P1 Aspartate 294 replaced by Glutamic acid to alter the inhibition spectrum [6]–[8]. Eluted proteins were judged >90% pure by SDS-12% PAGE, silver staining and immunoblotting. Serp-2 and CrmA were tested for Casp 1 and GzmB inhibitory activity, CrmA displaying greater (>5–6 fold) Casp 1 inhibition than Serp-2 (data not shown), as previously reported [6]–[9].

Serp-2 and CrmA were labeled with Fluorescein Isothionate (Fluorotag FITC conjugation kit, Sigma-Aldrich Canada Ltd., Mississauga, Ontario) and passed through G-25M gel filtration column to separate unbound FITC. The F/P (FITC/protein) ratio was 2.2 and 2.1, for Serp-2 and CrmA respectively. The caspase 1 inhibitory activity of FITC labeled proteins was assayed, displaying normal activity. BSA was labeled in parallel with FITC and used as control in entry assays.

### Enzyme activity analyses

For whole arterial lysates Serp-2, CrmA, 294A, or 294E treated rat femoral arteries (2–3 cm length) were excised at 0, 12, and 72****hours after angioplasty injury. The tissues were homogenized, lysed and extracted in 1mM EDTA buffer, centrifuged at 10,000****rpm, 8°C for 10****min to remove un-dissolved solids and supernatant stored at 80°C. For cell lysates, 1×10^7^/1****ml volume (HUVEC, THP-1, or T-cells) were treated with saline or apoptosis actuators (CPT, 2 µM for THP-1 and 10 µM for T cells; STS 0.5 µM from Sigma, Oakville, ON, Canada; or FasL, 3****ng/mL from Upstate solutions, Charlottesville, VA, U.S.A.), and each actuator given in combination with either Serp-2, CrmA, 294A, or 294E (500****ng/ml). Cells were collected at 6 hours, washed with cold saline and treated with 60 µl lysis buffer (150****mM NaCl, 20****mM Tris base, 1% (v/v) Triton-X 100 at pH 7.2, for 10****min, 4°C) followed by centrifugation at 10,000****rpm for 10****min, 8°C. Supernatant was collected and stored at −80°C until use. Protein concentration was measured (Bio-Rad Protein assay, Bio-Rad Laboratories, Hercules, CA, U.S.A.).

A subset of T cell cultures were treated with phorbol myristic acid (PMA, 1****ug/mL) and Ionophore A23187 (1****µg/mL) to induce a CTL-like (cytotoxic T lymphocyte) state. Medium from treated T cell cultures containing GzmB and perforin was removed after 2 hours incubation and applied to fresh, untreated T cell cultures together with Serp-2, CrmA, or D294A or E, with and without antibody to GzmB or perforin (Sigma) or the cell permeable small molecule inhibitor, ZAAD-CMK (ZAAD-chloromethylketone, Calbiochem, CedarLane, Hornby, ON), incubation for 12 or 24****hours [44].

To analyze cell death ELISA assay, fragmented nucleosomes were detected using quantitative sandwich-enzyme immunoassay as per the manufacturer's directions (Cell Death ELISA kit, Roche Diagnostics, Germany) with conjugated peroxidase measured photometrically at 405 nm with ABTS (2,2Ã–azino-di [3]-ethylbenzthiazoline sulfonate) as substrate using a Multiscan Ascent Spectrophotometer (Thermo LabSystems Inc., Beverly, MA, US).

For the DEVDase (Casp 3 and 7), 10****µl of the cell/tissue lysate was incubated at 37°C for one hour in 90****µl of reaction buffer (100****mM Acetyl-DEVD(Asp-Glu-Val-Asp)-AFC(7-amino-4-trifluoromethylcoumarin) (Bachem, Torrance, CA, U.S.A.), 100 mM HEPES, 0.5****mM EDTA, 20% (v/v) Glycerol and 5****mM DTT, pH 7.5). For IEPDase, 10****µl of the cell /tissue lysate was incubated at 37°C for one hour in 90 µl of reaction buffer (100****mM Acetyl-IEPD(Ile-Glu-Pro-Asp)-AFC(7-amino-4-trifluoromethylcoumarin) (Kamiya Biomedical Company, Seattle, WA), 50 mM HEPES, 0.05% (w/v) CHAPS, 10% (w/v) Sucrose and 5****mM DTT, pH 7.5) [35], [41], [42]. For the Cathepsin K, S, L, and V assays, 10 µl of the cell /tissue lysate was incubated in 90 µl of reaction buffer (5 mM Rhodamine 110, bisCBZ-L-Phenylalanyl-L-arginine amide, 50 mM Sodium acetate, 1 mM EDTA and 4****mM DTT, pH5.5) (Bachem, Torrance, CA, U.S.A.) [39]. For DEVDase and IEPDase hydrolyzed fluorochrome, 7-amino-4-trifluoromethyl coumarin was measured using a Spectrofluorometer (Fluoroskan; with excitation 405****nm, emission 527****nm) (Thermo LabSystems Inc., Beverly, MA, US) [39]. For the Cathepsin assay hydrolyzed fluorochrome, Rhodamine110 was measured using excitation 485****nm, emission at 527****nm. Final values are corrected for protein concentration. All these experiments were performed in triplicate, with three separate experiments performed for each condition, and the arbitrary fluorescent units from each reading used to derive the mean ± standard error for individual treatments and presented in the figures as bar graph with error bars indicating standard error.

### Statistical Analysis

The apoptotic enzyme activity measurement results unless otherwise mentioned represents mean ± SE from 3 to 5 replicates for each experiment. Significance was assessed by analysis of variance (ANOVA) with secondary Fishers least significant difference and Mann Whitney; p values <0.05 considered significant.

## Supporting Information

Figure S1 **Viral cross-class serpins alter Staurosporine-induced apoptotic responses in T cells and monocytes,** ***in vitro*** **.** Apoptotic responses were induced in T cells and monocytes using staurosporine. The ability of Serp-2, CrmA, or Serp-2 mutants to counteract this induction was measured by granzyme B and caspase 8 activity by IEPDase activity. Serp-2, but not CrmA nor D294A and D294E treatment of Jurkat T cells reduced caspase 8 and Granzyme B activity after staurosporine (STS) (**A**, p≤0.001) apoptosis actuator treatment. In THP-1 human monocytes, no cross-class serpins significantly reduced granzyme B or caspase 8 activity (**B**). The results shown here represent mean ± SE from 3 to 5 replicates for each experiment. Significance was assessed by analysis of variance (ANOVA) with secondary Fishers least significant difference and Mann Whitney analysis.
(TIFF)
