# Supplementary figures and images for: Viral Cross-Class Serpin Inhibits Vascular Inflammation and T Lymphocyte Fratricide; A Study in Rodent Models In Vivo and Human Cell Lines In Vitro
Source: PLoS One. 2012 Sep 26;7(9):e44694. doi: 10.1371/journal.pone.0044694 (PMC3458838; doi:10.1371/journal.pone.0044694)

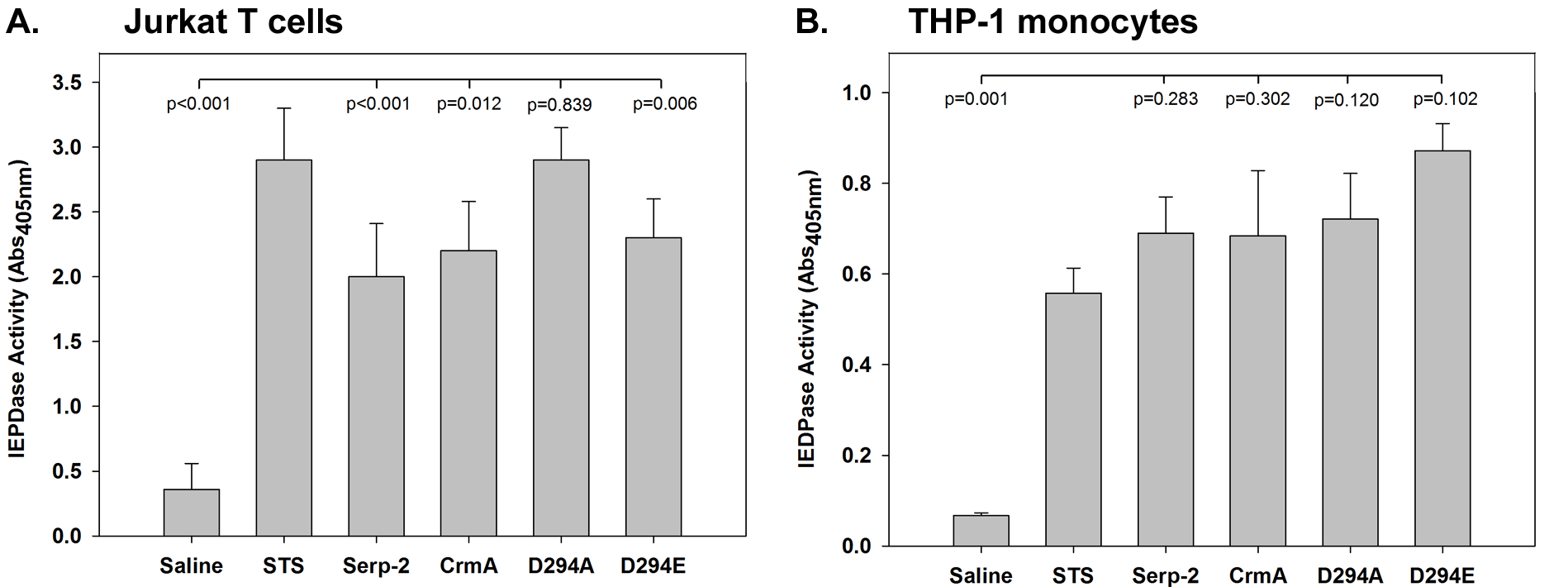

Supplement: Figure S1 — Viral cross-class serpins alter Staurosporine-induced apoptotic responses in T cells and monocytes, in vitro . Apoptotic responses were induced in T cells and monocytes using staurosporine. The ability of Serp-2, CrmA, or Serp-2 mutants to counteract this induction was measured by granzyme B and caspase 8 activity by IEPDase activity. Serp-2, but not CrmA nor D294A and D294E treatment of Jurkat T cells reduced caspase 8 and Granzyme B activity after staurosporine (STS) (A, p≤0.001) apoptosis actuator treatment. In THP-1 human monocytes, no cross-class serpins significantly reduced granzyme B or caspase 8 activity (B). The results shown here represent mean ± SE from 3 to 5 replicates for each experiment. Significance was assessed by analysis of variance (ANOVA) with secondary Fishers least significant difference and Mann Whitney analysis. (TIFF) [file pone.0044694.s001.tiff]
